# Dr. Roberts’ Case of Fatal Hæmorrhage from the Extraction of a Tooth; with the Treatment

**Published:** 1842-05-28

**Authors:** W. A. Roberts

**Affiliations:** Dentist and Surgeon, Edinburgh


*A case of fatal hemorrhage from the extraction of a tooth ; with the treatment.* By W. A. Roberts, Dentist and Surgeon, Edinburgh.—Mr. C.

Pen, of middle age, rather full in body, called upon me on Sunday, the 19th Dec., 1841, requesting to have a tooth removed that had given some uneasiness for a length of time. Upon examination, I found the dens sapientiæ of the right side of the lower jaw loose, the crown quite gone, and removed it without difficulty with a pair of forceps generally used for extracting the temporary teeth of children; it had three small fangs, the anterior one being the longest; the hæmorrhage nothing more than usual, and had ceased ere he left; the alveolus being plugged with lint, wetted with the camphorated spirit of wine. At half-past four of the same day, Mr. P. called again, the blood flowing in a continued stream, evidently from the anterior alveolus: cleaned it out from the bottom, and filled it up firmly with a strip of lint, pressed down with a curved instrument; when full, applied a compress of cork fitted to the part, and pressed upon firmly by the dens sapientiæ of the upper jaw, being likewise securely bandaged. Ordered astringent lotions, &c. The hæmorrhage was again checked, the saliva coming away unsustained. At this visit the patient informed me that he had had a tooth taken out a few years ago, which was followed by considerable bleeding for nearly three days, but was arrested by the application of caustic; as also, that his gums had bled to a great extent, and for a fortnight at a time. Of all this I was unfortunately ignorant until after three hours had elapsed from the removal of the stump. There was nothing indicating any hæmorrhagic tendency at the time I saw him first; and being a stranger to me I was consequently not acquainted with the history of his habit of body. I was sent for early on the Monday morning, and found the hæmorrhage had continued without intermission through the night; he had deferred sending for me unfortunately, as I had requested, supposing the bleeding would stop of itself. I found no coagulum about the mouth, or in what had been spat out, as in ordinary cases of hæmorrhage, the alveolus being as clear as when the root was first taken out. I put a piece of lunar caustic, the size of a pin's head, into the bleeding alveolus, pressed it down, and plugged with sponge lint, and bandage as formerly. The bleeding was once more stopped. Lotions of kino and alum were used with benefit. For more than an hour after this all appeared safe. In the course of the day Dr. Hay, of Queen street, the family medical attendant, called, and found the case as bad as ever. Dr. H. applied the actual cautery without benefit; attributing this circumstance to the instrument used, the first thing at hand being too thick at the point, I followed up Dr. Hay's suggestion a few hours afterwards, and used an iron better adapted to reach the bleeding vessel, but with no good result; during the operation the patient started, by which the iron slightly burnt his under lip (and here I may mention, the blood continued to ooze freely from this wound for several days.) Our success was various until Wednesday, the 22d; and on that day, if anything, the hæmorrhage was worse, and more alarming symptoms present: great sinking, weak pulse, giddiness, &c.; had serious thoughts it would be necessary to take up the carotid artery. Towards evening an improvement took place; the bleeding being once more under command by pressure; mild purgatives given, in consequence of a considerable quantity of blood having been swallowed. On Thursday, at 2 A. M., was sent for, as the patient had sunk to an alarming degree; Dr. Hay and myself attended immediately; found him recovering from a fainting fit; port wine was given; he rallied. Upon examination, found there was no active hæmorrhage from the original source, nor was there any afterwards. On this day, Mr. Nasmyth, of George street, saw the case, which was going on favourably, with the exception of a smart oozing from the gums and nostrils, which commenced after the bleeding from the alveolus had ceased. Upon the removal of the bandages, the face was much swollen and discoloured, from the effusion of blood into the cellular tissue, giving all the appearance of the result of a blow. From the 23d to the 27th still improving; pulse good; countenance less anxious; getting a quiet sleep occasionally; the sloughs drying up under the use of turpentine, &c., with no increase of hæmorrhage; mild aperients ordered, a little wine (port and claret,) and the use of tonics. For several days still gaining ground: the oozing from the gums being occasionally troublesome, a solution of the nitrate of silver was painted over them with advantage. On Monday, the 27th, Dr. Hay and Mr. Nasmyth considered it unnecessary to continue our meetings as we had done, but to see him occasionally, Dr. H. taking the charge of the case. From the 27th until the 30th, much the same; no active hæmorrhage. I had not seen the case for two days, when Dr. Hay informed me that a change for the worse had taken place, all the old symptoms being aggravated by a severe pain all over the mouth and head. Mr. Nasmyth and myself saw the patient on Sunday, the 2d of January, 1842: general depression with slight hæmorrhage from the gums, nostril, and the alveolus; pulse irregular. Claret given every two hours. The gums were washed with strong astringents. From the 2d to the 9th no improvement. Dr. Abercromby, York Place, was consulted. From the 9th gradually sinking. Wine given freely. The gums were touched with the proto-nitrate of mercury by Mr. Nasmyth, which only checked the hæmorrhage for a short time; they were turgid, of a purple colour, and almost covered the teeth; the features collapsed, cheek still discoloured, and all the symptoms of the disease, purpura hæmorrhagica, more decided. Although all was done that such eminent men could be expected to do, death put an end to this painfully interesting case on the following Tuesday, being three weeks and two days in duration.

In the course of my practice I have met with several cases of severe hæmorrhage following the extraction of a tooth, but always succeeded, by pressure, in checking it. One case in particular, the hæmorrhage was alarming, and had continued for two days. Upon examining the mouth, I discovered a portion of the alveolar process that had been splintered; upon taking this away, and removing the clot of blood, which nearly filled the mouth, and, in fact, was acting as a poultice, and also washing out the bleeding alveolus with warm water, I cut a small piece of sponge lint to the size of the cavity, and pressed firmly down with lint; over that a compress of cork, and securely bandaged, the hæmorrhage was effectually arrested. The heat of the mouth softens the wax, the sponge expands, and being confined, must of necessity press upon the mouth of the bleeding vessel. I have occasionally tried replacing the tooth with lint wrapped round the fangs, but never could depend upon it, but should think it would answer well with any of the single-rooted teeth, or the bicuspides; I never had occasion to try it in any of these teeth. In passing I may remark, that in all the cases that have come under my notice, I never saw the application of the actual cautery of much service; still, in extreme cases we are bound to employ it.—*Lond. Med. Gaz.* Feb. 11th, 1842.

[In the American Journal of the Medical and Physical Sciences, for the year 1828, we have given the history of a case of similar nature with the above, but of far more unfavourable character; as the patient was one of the "bleeders," or persons liable, by hereditary diathesis, to violent hæmorrhage from trivial causes. The paper containing the case referred to, brought up the knowledge extant upon the subject of hereditary hæmorrhage as nearly as possible to the day of publication. Though three or four communications of novel observations have since been presented in the European and American Journals, we do not recollect that any new therapeutic fact of importance has been elicited. It is therefore to be regretted that Mr. Roberts was unacquainted with the contents of our paper, as it contained two suggestions of high practical importance: 1st, The separation of the jaws by a block of wood, to prevent suction, as suggested by Dr. Physick, who met us in consultation on the case; and, 2d, The internal use of opiates, by the use of which for the relief of other symptoms, we completely arrested the hæmorrhage and saved the patient. Actual cautery and plugging of all kinds were fully proved to be unavailing in extreme cases, and from what we have seen more recently of capillary hæmorrhage, we cannot avoid the impression that the use of opium and the block might might have saved the life of the Edinburgh patient. The powder of alum plugged into the socket of a bleeding tooth is a more powerful styptic than the actual cautery in such situations.

As the paper to which we have referred has been published at length in several of the leading Parisian and German Journals, and fully noticed in those of London, we are rather surprised that no notice of the employment of opiates in capillary hæmorrhage has since attracted our attention.]

